# Current status of childhood interstitial lung disease in Turkiye: 5-year data from ChILD-TR

**DOI:** 10.1136/bmjresp-2026-004626

**Published:** 2026-11-02

**Authors:** Handan Kekeç, Ayse Tana Aslan, Tugba Sismanlar Eyuboglu, Tuğçe Ünlü, Sinem Can Oksay, Yasemin Mocan Çağlar, Özge Ülgen, Fatma Nur Ayman, Fatih Ercan, Gökçen Ünal, Çiğdem Korkmaz, Azer Kılıç Başkan, Abdulhamit Çollak, Ugur Ozcelik, Deniz Dogru, Raziye Atan, Figen Gülen, Bahar Girgin Dindar, Ece Halis, Esin Gizem Olgun, Fazılcan Zirek, Suat Fitöz, Meltem Kürtül Çakar, Sanem Eryilmaz Polat, Dilber Ademhan Tural, Ayşen Bingöl, Abdurrahman Erdem Başaran, Betül Bankoğlu Parlak, Ela Erdem Eralp, Bulent Karadag, Pınar Ergenekon, Mehmet Köse, Ali Ersoy, Melih Hangül, Pelin Asfuroğlu, Erkan Cakir, Hakan Yazan, Beste Ozesen, Erdem Gönüllü, Zeynep Seda Uyan, Ece Ocak, Aslı İmran Yılmaz, Cansu Yılmaz Yegit, Halime Nayır Büyükşahin, Gökçen Kartal Öztürk, Mahir Serbes, Tugba Ramasli Gursoy, Mina Gharibzadeh Hizal, Gözde Cavıldak Karaaslan, Gizem Özcan, Ali Özdemir, Sevgi Pekcan, Ayşe Ayzıt Kılınç Sakallı, Yasemin Gökdemir, Güzin Cinel, Nazan Cobanoglu, Saniye Girit, Nagehan Emiralioğlu, Ebru Yalcin, Berna Oğuz, Diclehan Orhan, Ahmet Cevdet Ceylan, Nural Kiper

**Affiliations:** 1 Department of Pediatric Pulmonology, Gazi University Faculty of Medicine, Ankara, Turkey; 2 Department of Pediatric Pulmonology, Medeniyet University Faculty of Medicine, Istanbul, Turkey; 3 Department of Pediatric Pulmonology, Istanbul Medeniyet University, Istanbul, Türkiye; 4 Department of Pediatric Pulmonology, Meram Faculty of Medicine, Necmettin Erbakan University, Konya, Türkiye; 5 Department of Pediatric Pulmonology, Istanbul University-Cerrahpasa, Cerrahpasa Faculty of Medicine, Istanbul, Türkiye; 6 Department of Pediatric Pulmonology, Hacettepe University Faculty of Medicine, Ankara, Turkey; 7 Department of Pediatric Pulmonology, Ege University Faculty of Medicine, Izmir, Turkey; 8 Department of Pediatric Pulmonology, Ankara University Faculty of Medicine, Ankara, Turkey; 9 Department of Radiology, Ankara University Faculty of Medicine, Ankara, Turkey; 10 Department of Pediatric Pulmonology, Ankara Bilkent City Hospital, Ankara, Turkey; 11 Department of Pediatric Pulmonology, Akdeniz University Faculty of Medicine, Antalya, Turkey; 12 Department of Pediatric Pulmonology, Marmara University Faculty of Medicine, Istanbul, Turkey; 13 Department of Pediatric Pulmonology, Erciyes University Faculty of Medicine, Kayseri, Turkey; 14 Department of Pediatric Pulmonology, Cengiz Gökçek Maternity and Children’s Hospital, Gaziantep, Turkey; 15 Department of Pediatric Pulmonology, Istinye University Faculty of Medicine, Istanbul, Turkey; 16 Department of Pediatric Pulmonology, Medipol University Faculty of Medicine, Istanbul, Turkey; 17 Department of Pediatric Pulmonology, Dokuz Eylül University Faculty of Medicine, Izmir, Turkey; 18 Department of Pediatric Pulmonology, Koç University Faculty of Medicine, Istanbul, Turkey; 19 Department of Pediatric Pulmonology, Izmir City Hospital, Izmir, Turkey; 20 Department of Pediatric Pulmonology, Konya City Hospital, Konya, Turkey; 21 Department of Pediatric Pulmonology, Başakşehir Çam and Sakura City Hospital, Istanbul, Turkey; 22 Department of Pediatric Pulmonology, Mardin Training and Research Hospital, Mardin, Turkey; 23 Department of Pediatric Pulmonology, Behçet Uz Training and Research Hospital, Izmir, Turkey; 24 Department of Pediatric Allergy and Immunology, Çukurova University Faculty of Medicine, Adana, Turkey; 25 Department of Pediatric Pulmonology, Van Training and Research Hospital, Van, Turkey; 26 Department of Pediatric Pulmonology, Ankara Training and Research Hospital, Ankara, Turkey; 27 Department of Pediatric Pulmonology, Kayseri City Hospital, Kayseri, Turkey; 28 Department of Pediatric Pulmonology, Mersin City Hospital, Mersin, Turkey; 29 Department of Radiology, Hacettepe University Faculty of Medicine, Ankara, Turkey; 30 Department of Pathology, Hacettepe University Faculty of Medicine, Ankara, Turkey; 31 Department of Medical Genetics, Ankara Bilkent City Hospital, Ankara, Turkey

**Keywords:** Rare lung diseases

## Abstract

**Introduction:**

Childhood interstitial lung disease (chILD) encompasses various diffuse parenchymal lung diseases (DPLDs) in children. We aimed to assess the current status and temporal changes of chILD in Türkiye using updated data from the chILD-Türkiye (chILD-TR) registry.

**Method:**

By May 2025, all patients registered in the chILD-TR were included in the study. Patients with missing data were excluded. Patients were divided into two groups: disorders primarily appearing in infancy (DPLD-A) and disorders that can occur at any age (DPLD-B). Clinical and demographic data were compared between the groups.

**Results:**

711 patients (44.7% female) were included. The registry-based prevalence estimate of chILD in Türkiye was approximately 30–35 cases per million children. The current age was 135 (16-383) months. The time from first symptoms to admission was 3 (0–217) months. Age at diagnosis was 59 (11-273) months. Common symptoms were cough (59.5%) and dyspnoea (35.4%). 136 patients (19.1%) required oxygen. 25 patients (3.5%) died. Among patients, 126 (17.7%) had DPLD-A, 549 (77.2%) had DPLD-B and 36 (5.1%) had uncategorised disorders. The DPLD-A group was younger and had earlier symptom onset and older diagnosis age (p<0.05). Growth z-scores were lower in the DPLD-A group (p<0.05). Parental consanguinity and mechanical ventilation use were higher in the DPLD-A group (p<0.05). No differences in spirometry and diffusing capacity of the lungs for carbon monoxide were found between DPLD-A and DPLD-B (p>0.05). Chest CT features varied: rates of ground-glass opacity and pectus excavatum were higher in the DPLD-A group, whereas nodules, peribronchial thickening, mosaic perfusion and bronchiectasis were more predominant in the DPLD-B group (p<0.05).

**Conclusion:**

This study shows that children with DPLD-A are diagnosed later despite earlier symptoms and growth issues, revealing a recognition gap and highlighting the need for early genetic screening and support. In DPLD-B, airway findings emphasise the need for long-term monitoring. These insights support phenotype-based diagnosis and treatment to improve outcomes in children with chILD.

**WHAT IS ALREADY KNOWN ON THIS TOPIC**

**WHAT THIS STUDY ADDS**

- Updated data from the nationwide chILD-Türkiye (chILD-TR) registry, including 711 children, demonstrate substantial phenotypic diversity and distinct clinical and radiological characteristics between DPLD-A and DPLD-B, with DPLD-A characterised by earlier symptom onset but older age at diagnosis, greater growth impairment and higher mechanical ventilation requirements.

**HOW THIS STUDY MIGHT AFFECT RESEARCH, PRACTICE OR POLICY**

- The diagnostic gap identified in DPLD-A and the distinct phenotypic patterns captured by the chILD-TR registry highlight the importance of earlier recognition, timely genetic evaluation and phenotype-based diagnostic and long-term management strategies.

## Introduction

 Childhood interstitial lung disease (chILD) is a group of rare, complex and chronic respiratory conditions that affect infants, children and adolescents. This diverse range of diffuse parenchymal lung disease (DPLDs) shows significant variation in causes, clinical signs, imaging results and outcomes. ChILD primarily affects lung tissue and is often associated with widespread abnormalities in the alveolar and interstitial spaces, leading to impaired gas exchange and declining respiratory function.^1^ The disease can progress from mild to severe, life-threatening respiratory failure, especially in young children. Currently, over 200 specific clinical conditions are classified under chILD, including developmental disorders, genetic and surfactant issues, immune system abnormalities, environmental influences and systemic diseases. This high diversity makes diagnosis, classification and treatment challenging, often requiring a multidisciplinary and personalised approach.^2^

Over the past 20 years, the development of national and international chILD registries has greatly enhanced understanding of chILD, particularly in areas such as epidemiology, disease types and clinical variability. These registry efforts have enabled the systematic collection of data from large patient populations, making it easier to identify rare disease entities more accurately and better estimate their true impact. As awareness of chILD has increased among clinicians and as advances in imaging, genetics and diagnostic algorithms have occurred, registry frameworks have been continually expanded and improved, supporting earlier diagnosis and more consistent classification of chILD across healthcare systems.^3–6^ Despite progress in imaging, genetics and molecular testing, chILD remains difficult to diagnose, and knowledge about its prevalence, natural progression and long-term outcomes is still limited, especially in children.

The chILD-Türkiye (chILD-TR) registry is a multicentre, nationwide collaboration that collects data from tertiary paediatric pulmonology centres across Turkey, providing a representative overview of chILD in the country. This study aimed to evaluate the current epidemiological and clinical characteristics of chILD in Türkiye, assess changes over time and compare children with disorders primarily appearing in infancy (DPLD-A) with those with disorders that can occur at any age (DPLD-B), using updated data from the chILD-TR registry.

## Methods

This multicentre, retrospective cohort study used data from chILD-TR. As of May 2025, all patients registered in the chILD-TR were included, except those with missing essential data. The chILD-TR was established in November 2021^7^ and collected clinical and demographic data, including sex, current age, age at symptom onset, age at diagnosis, follow-up duration, neonatal history, parental consanguinity, presenting symptoms, growth z-scores, physical exam findings and radiological results.

Functional respiratory data included spirometry and diffusing capacity of the lungs for carbon monoxide (DLCO) measurements. Pulmonary function tests (PFTs) were performed in accordance with the standardised acceptability criteria established by the American Thoracic Society and European Respiratory Society (ATS/ERS) for paediatric populations.^8^ The DLCO was measured using the single-breath method. Values between the 5th and 95th percentiles were considered within the normal reference range.^9^ All spirometry and DLCO assessments were performed at the time of chILD diagnosis. Percent-predicted spirometry and DLCO values were recorded as calculated and reported by the participating centres. Information on the specific reference equations used to calculate these values was not collected in the registry.

Additional information included genetic analysis results, use of supplemental oxygen, history of mechanical ventilation, neonatal intensive care unit admission, pulmonary hypertension (PH), haematopoietic stem cell transplantation, lung biopsies and vital status (alive or deceased). PH assessment in children with chILD included annual transthoracic echocardiography performed by paediatric cardiologists to screen for PH. PH was defined as a mean pulmonary arterial pressure >20 mmHg and a pulmonary vascular resistance >2 Wood Units.^10 11^ When echocardiographic findings suggested possible PH, confirmation was obtained by measuring pulmonary artery pressure during cardiac catheterisation. A lung biopsy was performed when histopathologic confirmation was needed.

Patients were divided into the DPLD-A and DPLD-B groups, and their demographic, clinical and radiological characteristics were compared.

### Patient and public involvement

This was a retrospective registry-based study. Patients and the public were not involved in the design, conduct, reporting or dissemination plans of this research.

### Classification of ChILD

All patients with a confirmed chILD diagnosis meeting established international criteria were enrolled in the chILD-TR registry.^7^ On registration, patients were systematically classified using the DPLD classification developed by the multicentre chILD-EU network.^5 12 13^ Accordingly, patients were categorised into two main groups: DPLD-A and DPLD-B. The diagnostic subcategories within each group are summarised in table 1 (online supplemental file 1).

**Table 1:** Classification of DPLD subgroups and diagnostic categories in the chILD-TR registry

DPLD group		
Disorders primarily appearing in infancy (DPLD-A)	DPLD-A1	Diffuse developmental disorders
	DPLD-A2	Growth abnormalities/deficient alveolarisation
	DPLD-A3	Infant conditions of undefined aetiology
	DPLD-A4	Disorders related to the alveolar surfactant region
	DPLD-Axe	Unclear RDS in the mature neonate
	DPLD-Ay	Unclear RDS in the almost mature neonate (30–36 weeks)
Disorders that can occur at any age (DPLD-B)	DPLD-B1	Disorders related to systemic disease processes
	DPLD-B2	Disorders in the presumed immune-competent host related to exposures (infectious/non-infectious)
	DPLD-B3	Disorders in the immunocompromised host or transplant recipient
	DPLD-B4	Disorders related to structural processes of the pulmonary vessels
	DPLD-B5	Disorders related to reactive lymphoid lesions
	DPLD-Bx	Unclear RDS in the non-neonate

chILD-TR registry, childhood interstitial lung disease in Turkiye registry; DPLD-A, disorders primarily appearing in infancy; DPLD-B, disorders that can occur at any age; RDS, respiratory distress syndrome .

### Statistical analysis

All statistical analyses were performed using IBM SPSS Statistics V.22.0 (IBM Corp., Armonk, NY, USA). Descriptive statistics included frequencies and percentages for categorical variables and means±SD or medians with ranges (minimum–maximum) for continuous variables. Associations between categorical variables were assessed using Pearson’s χ2 or Fisher’s exact test, as appropriate. For comparisons between two independent groups, the Mann–Whitney U test was used for non-normally distributed continuous variables, whereas the independent-samples t-test was used for normally distributed continuous variables. p<0.05 was considered statistically significant.

## Results

A total of 748 patients were registered; 37 with missing data were excluded, leaving 711 patients (44.7% female) for analysis. The study’s flow diagram is presented in figure 1. Based on national population data showing that children constitute 25.5% of Türkiye’s population (approximately 21.8 million) and on the 748 patients recorded in the chILD-TR registry, the registry-based prevalence estimate was approximately 30–35 cases per million children.

**Figure 1:**
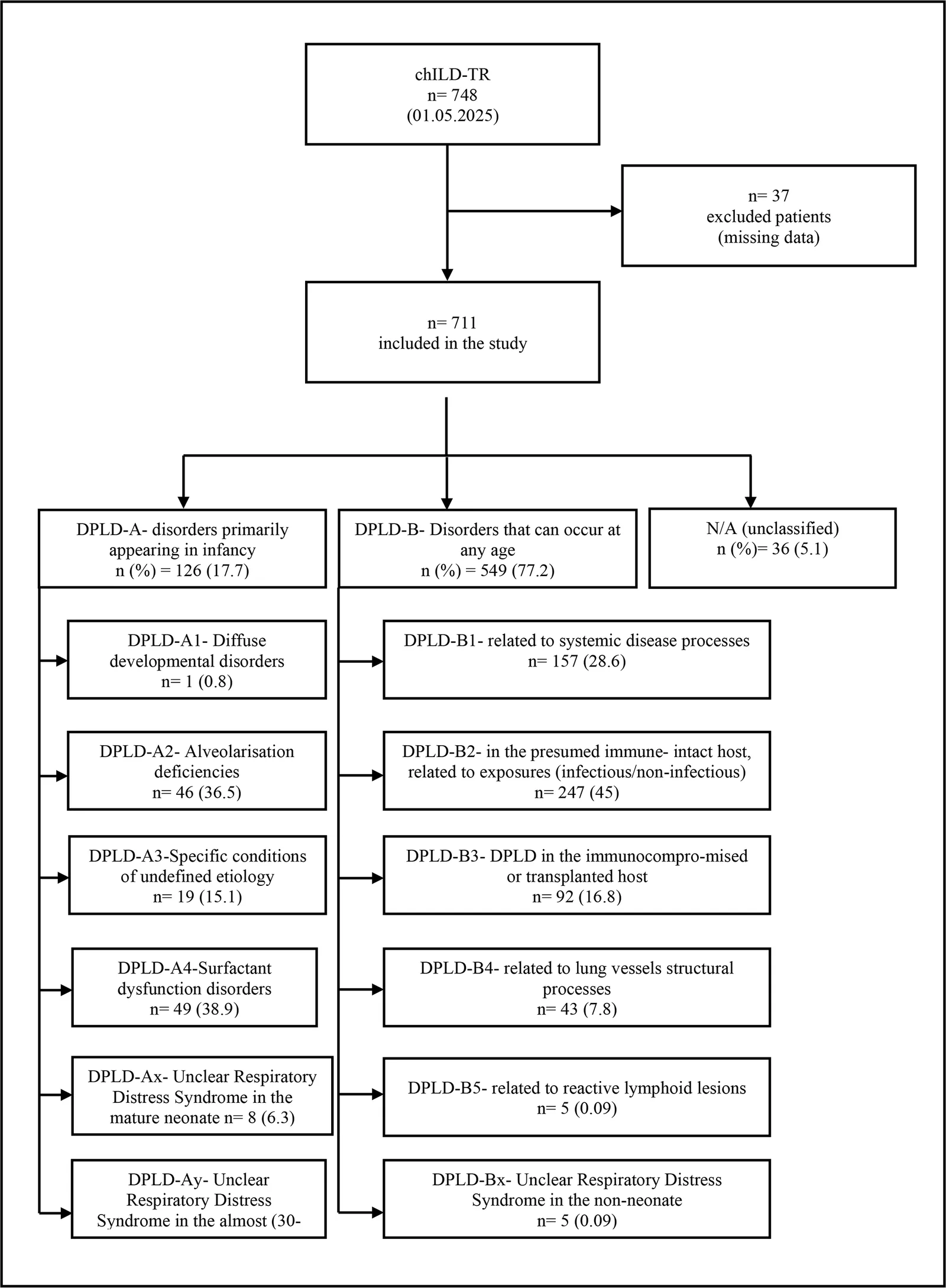
Flow diagram of the study. chILD-TR registry, childhood interstitial lung disease in Turkiye registry; DPLD-A, disorders primarily appearing in infancy; DPLD-B, disorders that can occur at any age.

Current age was 135 (range, 16–383) months. The time from first symptoms to initial admission was 3 (range, 0–217) months. The age at chILD diagnosis was 59 (range, 11–273) months. The most common presenting symptoms were cough (59.5%) and dyspnoea (35.4%). A total of 136 patients (19.1%) required oxygen. 67 (9.4%) patients had a history of haematopoietic stem cell transplantation. Feeding disorders were reported in 95 (13.4%) patients, gastro-oesophageal reflux in 67 (9.4%) and a history of aspiration in 19 (2.7%). Gastrostomy was performed in 9 (1.3%) patients. Regarding diagnostic methods, chest CT was performed in 664 (93.4%) patients, bronchoscopy in 300 (42.2%), genetic analyses in 204 (28.7%), lung biopsy in 65 (9.1%) and chest MRI in 12 (1.7%). A total of 25 patients (3.5%) died. The demographic and clinical data are presented in table 2.

**Table 2:** Demographic and clinical data of all patients

	n=711 (%)
Female	318 (44.7)
Male	393 (55.3)
Current age (months)	135 (16–383)
Age at chILD diagnosis (months)	59 (11–273)
Age of the first symptoms related to chILD (months)	58 (0–299)
Time between the first symptom and the diagnosis of chILD (months)	3 (0–271)
Weight z-score	−0.88 (−8.80-3.62)
Height z-score	−0.63 (−8 to 3.55)
Body mass index z-score	−0.51 (−8.90 to 3.37)
Symptoms	
Cough	423 (59.5)
Dyspnoea	252 (35.4)
Tachypnoea	246 (34.6)
Wheezing	246 (34.6)
Fever	56 (7.9)
Cyanosis	46 (6.4)
Chest pain	39 (5.5)
Haemoptysis	35 (4.9)
Oxygen support	136 (19.1)
Mechanical ventilation	46 (6.8)
Invasive mechanical ventilation	8 (1.1)
Non-invasive mechanical ventilation	38 (5.8)
Feeding disorders	95 (13.4)
Gastro-oesophageal reflux	67 (9.4)
History of aspiration	19 (2.7)
Gastrostomy	9 (1.3)
Pulmonary hypertension	38 (5.3)
Haematopoietic stem cell transplantation	67 (9.4)
Diagnostic methods	
Chest CT	664 (93.4)
Bronchoscopy	300 (42.2)
Genetic analyses	204 (28.7)
Lung biopsy	65 (9.1)
Chest MRI	12 (1.7)
Deceased patients	25 (3.5)

chILD, childhood interstitial lung disease .

The most common treatment options included oral steroids in 282 (39.6%) patients, inhaled steroids in 270 (37.9%), azithromycin in 166 (23.3%), pulse steroids in 140 (19.7%) and hydroxychloroquine in 51 (7.1%). The treatments for all patients are shown in table 3.

**Table 3:** Treatment of patients

Treatments	N (%)
Oral steroid	282 (39.6)
Inhaled steroid	270 (37.9)
Azithromycin	166 (23.3)
Pulse steroid	140 (19.7)
Hydroxychloroquine	51 (7.1)
IVIG	49 (6.9)
Mycophenolate mofetil	16 (2.2)
Azathioprine	15 (2.1)
Whole lung lavage	6 (0.8)
Other treatments*	233 (32.7)

* Other treatments included targeted therapies (JAK inhibitors, anti-IL-1, anti-IL-6 and anti-TNF agents), bronchodilators, enzyme replacement therapies, antifibrotic agents and selected supportive treatments tailored to the underlying disease.

IL-1, interleukin-1; IL-6, interleukin-6; IVIG, intravenous immunoglobulin; JAK, Janus kinase inhibitors. ; TNF, tumour necrosis factor.

Of the patients, 126 (17.7%) had DPLD-A, 549 (77.2%) had DPLD-B and 36 (5.1%) had NA1 (uncategorised). No between-group differences were found in sex (p=0.286). The DPLD-A group was younger (p<0.001), had an earlier age at symptom onset (p<0.001) and had a higher age at chILD diagnosis (p=0.017) than the DPLD-B group. The weight z-score was −1.43 (−9.67 to 7.26), the height z-score was −1.04 (−7 to 9.43) and the BMI z-score was −1 (−9.30 to 2.26) in DPLD-A, all of which were significantly lower than those in the DPLD-B group (p<0.001, p=0.012 and p<0.001, respectively). Parental consanguinity (p=0.013) and mechanical ventilation use (p<0.001) were more common in the DPLD-A group, whereas history of haematopoietic stem cell transplantation was more frequent in the DPLD-B group (p=0.001). No differences in PFTs (n=132) or DLCO (n=68) (p>0.05) were found between the DPLD-A and DPLD-B groups. The comparison of the DPLD-A and DPLD-B groups is shown in table 4.

**Table 4:** Comparison of the disorders primarily appearing in infancy (DPLD-A) and disorders that can occur at any age (DPLD-B) groups by demographic and clinical data

	DPLD-A n=126 (%)	DPLD-B n=549 (%)	P
Female	51 (40.5)	251 (45.7)	0.286
Male	75 (595)	298 (54.3)	
Current age (months)	97 (16–328)	149 (19–383)	**<0.001**
Age at onset of first symptoms related to chILD (months)	8.5 (0–190)	75 (0–299)	**<0.001**
Age at chILD diagnosis (months)	68 (11–273)	56 (11–235)	**0.017**
Time between the first symptom and chILD diagnosis (months)	4 (0–164)	3 (0–271)	0.870
Weight z-score	−2.0 (−7.83 to 2.0)	−0.79 (−8.8 to 3.62)	**<0.001**
Height z-score	−0.99 (−7.0 to 3.55)	−0.59 (−8.0 to 3.52)	**0.004**
Body mass index z-score	−1.22 (−8.9 to 3.37)	−0.32 (−8.3 to 3.04)	**<0.001**
Parental consanguinity	55 (43.7)	176 (32.1)	**0.013**
History of NICU admission	73 (57.9)	104 (18.9)	**<0.001**
Oxygen support	46 (36.5)	82 (14.9)	**<0.001**
Mechanical ventilation	22 (17.5)	23 (4.2)	**<0.001**
Pulmonary hypertension	15 (11.9)	21 (3.8)	**<0.001**
Decased patients	10 (7.9)	13 (2.3)	**0.002**
Spirometry	n=10	n=122	
FEV1%	78 (48–101)	72 (16–135)	0.527
FVC%	73 (63–87)	75 (17–136)	0.708
FEF_25-75_%	100 (28–168)	78 (9–150)	0.079
DLCO	n=2	n=67	
	44 (36–53)	77 (21–159)	–

Bold p-values indicate statistical significance (p<0.05).

chILD, childhood interstitial lung disease ; DLCO, diffusion capacity of the lung; FEF₂₅–₇₅%, forced expiratory flow at 25%–75% of the forced vital capacity; FEV₁%, Forced expiratory volume in 1 second; FVC%, forced vital capacity; NICU, neonatal intensive care unit.

Comparison of the chest CT findings showed that ground-glass opacity (GGO) (p=0.001) and pectus excavatum (p<0.001) were more common in DPLD-A. Nodules or nodular opacities (p<0.001), peribronchial thickening (p=0.021), mosaic perfusion (p<0.001) and bronchiectasis (p<0.001) were more common in DPLD-B. The comparison of the radiologic findings is shown in table 5. Different radiologic patterns of DPLD-A and DPLD-B of chILD are presented in online supplemental files 2 and 3.

**Table 5:** Comparison of the disorders primarily appearing in infancy (DPLD-A) and disorders that can occur at any age (DPLD-B) by radiological data

	DPLD-A n=126 (%)	DPLD-B n=549 (%)	P
Ground-glass opacities	76 (60.3)	263 (47.9)	**0.001**
Linear/reticular opacities	34 (26.9)	140 (25.5)	0.103
Interseptal thickening	33 (26.2)	139 (25.3)	0.242
Peribronchial thickening	30 (23.8)	192 (34.9)	**0.021**
Focal consolidations	28 (22.2)	135 (24.6)	0.563
Increased lung aeration	27 (21.4)	136 (24.7)	0.208
Mosaic perfusion	26 (20.6)	243 (44.2)	**<0.001**
Cystic lesions	15 (11.9)	52 (9.4)	0.113
Nodule/nodular opacity	15 (11.9)	163 (29.6)	**<0.001**
Pectus excavatum	13 (10.3)	12 (2.1)	**<0.001**
Emphysema	11 (8.7)	44 (8.0)	0.637
Honeycombing	9 (7.1)	33 (6.0)	0.205
Mediastinal lymphadenopathy	6 (6.7)	92 (16.7)	**0.002**
Bronchiectasis	1 (0.07)	93 (16.9)	**<0.001**
Traction bronchiectasis	1 (0.07)	23 (4.1)	**<0.001**

Bold p-values indicate statistical significance at p<0.05

DPLD-A, disorders primarily appearing in infancy ; DPLD-B, disorders that can occur at any age .

## Discussion

This nationwide, multicentre, registry-based study provides an updated overview of chILD in Türkiye using data from the chILD-TR registry. Analysis of clinical, radiologic and treatment data from 711 children highlights the diversity of chILD and the complexity of these rare disorders in real-world practice. Results showed a significant clinical burden, phenotypic diversity and differences between the DPLD-A and DPLD-B groups. Children with DPLD-A, despite presenting with symptoms at an earlier age, had greater growth impairment and were diagnosed at an older age than those with DPLD-B. The DPLD-A group also had higher rates of parental consanguinity and mechanical ventilation, whereas a history of haematopoietic stem cell transplantation was more frequent in the DPLD-B group. Chest CT findings showed that GGO and pectus excavatum were more common in the DPLD-A group, whereas mosaic perfusion, nodules, lymphadenopathy and bronchiectasis were more common in the DPLD-B group.

Understanding the epidemiology of chILD, including its incidence and prevalence, remains challenging because these disorders are rare and highly heterogeneous. Previous studies have reported considerable variation in chILD prevalence across populations.^14–16^ A recent population-based study from Spain reported an average annual incidence of 8.18 new cases per million children and a prevalence of 46.53 cases per million children.^14^ Similarly, the estimated prevalence of chILD in France was 44 cases per million children.^4^ In contrast, the Australia and New Zealand Registry Network estimated a prevalence of approximately 1.5 cases per million children,^15^ while a study from the UK and Ireland reported a prevalence of 3.6 cases per million children aged 0–16 years.^16^

Based on national population data indicating that children constitute 25.5% of Türkiye’s population (approximately 21.8 million children) and on the 748 patients registered in chILD-TR, the registry-based prevalence of chILD was estimated at approximately 30–35 cases per million children.^17^ This estimate is lower than those reported in recent European cohorts from Spain and France,^4 14^ which is notable given Türkiye’s relatively high rate of consanguinity and its potential contribution to certain genetically determined forms of chILD. Although ChILD-TR has broad national coverage and includes data from all paediatric pulmonology centres across Türkiye, prevalence estimates should be interpreted in light of the inherent diagnostic challenges of chILD. The rarity and marked clinical and aetiological heterogeneity of these disorders, together with variable and sometimes nonspecific clinical presentations, may contribute to delayed recognition or underdiagnosis, particularly in less typical cases. Moreover, differences in case ascertainment, diagnostic practices, referral pathways, registry coverage, healthcare access and increased awareness of chILD over time can significantly affect these estimates. Additionally, access to paediatric pulmonology services is not uniformly available across all regions of Türkiye, which may have affected the detection and registration of chILD cases. Therefore, the lower registry-based estimate observed in Türkiye should not necessarily be interpreted as reflecting a lower underlying disease burden, and direct comparisons between countries should be made with caution.

chILD is marked by substantial diagnostic and phenotypic diversity, and recognition of this heterogeneity has improved with the development of registry networks and growing awareness of these rare disorders. Earlier studies included relatively small cohorts; Griese *et al* reported 38 children, of whom 55.3% were classified as DPLD-A and 44.7% as DPLD-B.^18^ The Australian and New Zealand registry, with 115 patients, showed a varied distribution, including 106 children aged ≤2 years.^15^ In Türkiye, the first ChILD-TR report, based on data available as of January 2023, included 370 patients from 19 centres, with 21.9% classified as DPLD-A and 78.1% as DPLD-B, and no unclassified cases.^7^ In the present study, the registry expanded to 711 patients from 27 centres; 17.7% were classified as DPLD-A, 77.2% as DPLD-B and 5.1% remained unclassified. The most recent data reflect increased awareness, improved case detection and greater recognition of subgroups. The expansion of the registry, together with advances in diagnosis and classification, has enabled recognition of rare and previously underrepresented phenotypes, shifts in subgroup distributions and the identification of unclassified cases. These developments reflect the evolving characterisation of the chILD spectrum in Türkiye and highlight the value of longitudinal registry data for improving our understanding of its epidemiological and phenotypic diversity and for supporting future research and clinical practice.

Establishing a definitive diagnosis of chILD remains challenging and often requires a multimodal approach integrating clinical, radiologic and molecular assessments. The distribution of diagnostic methods in our registry also reflects evolving strategies for diagnosing chILD.^19^ In the initial chILD-TR report,^7^ chest CT was performed in 92.4% of cases, bronchoscopy in 39.2%, genetic testing in 30.9% and lung biopsy in 14.3%. In the updated cohort, chest CT remained the most frequently used diagnostic method (93.4%), while bronchoscopy was performed in 42.2% of patients. Conversely, the proportion of patients undergoing lung biopsy decreased to 9.1%, and genetic analysis remained common at 28.7%. This trend aligns with current chILD diagnostic frameworks that emphasise multidisciplinary, stepwise evaluation, including clinical findings, chest CT, bronchoalveolar lavage and genetic testing.^19^ Recent advances in molecular diagnostics and increased awareness of monogenic chILDs may reduce the need for invasive procedures such as surgical lung biopsy, which is now reserved for cases where noninvasive tests fail. Although chest MRI has limitations in assessing certain abnormalities, it is a promising radiation-free option for paediatric patients. Our updated cohort included patients who underwent chest MRI, reflecting the growing use of chest MRI in chILD diagnosis.

A key strength of this study is the direct comparison between the DPLD-A and DPLD-B groups, which revealed clear, clinically meaningful differences. The DPLD-A group had significantly lower growth z-scores, indicating more severe impairment in growth and nutritional status than the DPLD-B group. These findings align with the initial report from the chILD-TR registry and further support the role of growth failure as a key feature of DPLD-A.^7^ As with other chronic lung diseases, growth parameters in chILD are crucial indicators of disease severity and overall health, often providing insights beyond lung function alone, particularly in younger children, for whom reliable PFTs are limited. Given the increased caloric demands associated with chronic lung disease, the direct impact of nutrition on lung growth and the ongoing development of the lungs through adolescence and into adulthood, sustained attention to nutrition and feeding practices throughout the lifespan is vital for patients with chILD, especially those with DPLD-A.^19–21^

This study found that children with DPLD-A presented with symptoms at an earlier age but were diagnosed at an older age than those with DPLD-B. They also had higher rates of consanguinity, neonatal intensive care unit admission, oxygen therapy, mechanical ventilation, PH and mortality, suggesting a greater clinical burden, systemic involvement and more severe disease progression in the DPLD-A group. Diagnostic delays in chILD may result from nonspecific symptoms, phenotypic overlap and the need for advanced genetic and multidisciplinary assessments. These findings underscore the importance of early suspicion, growth monitoring, prompt referral, genetic testing and timely respiratory support to improve management and outcomes.^20 22 23^

Pulmonary function testing is crucial for evaluating disease in children with chILD. Despite clinical differences, spirometric parameters showed no significant variation, likely reflecting chILD heterogeneity, in which different types affect lung function similarly. Ring *et al*^24^ noted the limited sensitivity of PFTs, especially in younger children and at early disease stages, which means that spirometric changes may not fully reflect severity or variability. Therefore, PFTs should be interpreted alongside clinical and radiological findings, not as standalone markers, during assessment and follow-up. Chest CT is crucial in diagnosing chILD, revealing disease phenotype and stage. Studies show CT patterns evolve with age: early childhood shows GGO, while later stages exhibit cystic, nodular and fibrotic changes.^25 26^ Our findings align with this: DPLD-A, mostly hereditary causes, shows inflammation in early stages before fibrosis becomes dominant, whereas DPLD-B indicates a more advanced stage with chronic remodelling. CT features such as peribronchial thickening, mosaic perfusion, nodules, bronchiectasis and traction bronchiectasis highlight involvement of the lungs and airways, as well as immune activity. These results underscore chest CT’s role in distinguishing phenotypes and guiding long-term management.

Therapeutic approaches in chILD vary widely and are influenced by disease subtype, underlying cause, age at presentation and centre expertise.^1 27^ Data from the French Respirare cohort (n=790, 42 centres) showed frequent use of corticosteroid pulse therapy (56%) and oral corticosteroids (44%), followed by azithromycin (27.2%), immunosuppressives (20.3%) and hydroxychloroquine (15.9%).^4^ US data indicated that up to 78% of patients received corticosteroids, with 57% receiving intravenous therapy and 10.5% receiving hydroxychloroquine.^27^ Similarly, Australian and New Zealand cohorts reported corticosteroids (59.6%), hydroxychloroquine (34.6%) and azithromycin (25%) as common treatments.^15^ In the first chILD-TR report, oral corticosteroids were used in 25.6%, inhaled corticosteroids in 16.4%, pulse therapy in 15.1%, immunosuppressives in 5.1% and hydroxychloroquine in 1.6%.^7^ In the updated cohort, corticosteroid use increased, with oral (39.6%) and inhaled (37.9%) corticosteroids, pulse therapy (19.7%), azithromycin (23.3%) and hydroxychloroquine (7.1%). These findings reflect evolving treatment practices and increased recognition of inflammatory and genetic phenotypes. Immunomodulatory agents (eg, intravenous immunoglobulin, mycophenolate mofetil and azathioprine) and targeted therapies (eg, JAK inhibitors, anti–interleukin (IL)-1, anti–IL-6, anti-tumour necrosis factor agents, T-cell and B-cell–directed therapies and calcineurin inhibitors) are used in selected cases based on disease mechanisms. Despite corticosteroid-based strategies remaining the mainstay, substantial inter-centre variability persists, underscoring the need for phenotype-driven, personalised, evidence-based treatment strategies and further research in chILD.^28–30^

Respiratory morbidity is vital for assessing disease severity and outcomes in children with chILD. The ATS guideline on home oxygen therapy for children with chronic respiratory illnesses strongly recommends prescribing oxygen therapy for chILD with severe chronic hypoxaemia.^31^ In the French Respirare Cohort, oxygen therapy was used in 52% of patients.^4^ In the Children’s Hospital of Philadelphia cohort of 306 patients, 32.7% required chronic home oxygen therapy, 12.4% received chronic non-invasive respiratory support and 4.6% required chronic invasive respiratory support via tracheostomy.^27^ Our findings showed variability in respiratory support needs among different groups, likely reflecting differences in disease spectrum, severity and clinical practices. Nearly one-fifth of patients (19.1%) needed long-term oxygen therapy, while smaller percentages required non-invasive (5.8%) or invasive respiratory support (1.1%), highlighting the chronic and potentially severe respiratory effects of the disease. Overall, these data suggest many children with chILD develop chronic respiratory failure, with some progressing to require more intensive respiratory support. The mortality rate of 3.5% further emphasises the life-threatening potential of chILD, even with modern supportive care.

The study has several limitations, including its retrospective design, the lack of genetic data for each patient and the small number of patients who underwent lung biopsies. Spirometry and DLCO measurements were available for only some patients because of age-related challenges and limited access to DLCO at certain participating centres. The reference equations used to calculate percent-predicted spirometry and DLCO values were not recorded in the registry. The registry-based prevalence estimate should also be interpreted cautiously. Despite the broad national coverage of chILD-TR, the rarity, clinical heterogeneity and diagnostic complexity of chILD may lead to delayed recognition or underdiagnosis, particularly in patients with atypical presentations, thereby underestimating the true disease burden. Additionally, some patients in the registry had already reached adulthood by the time of analysis, despite having been diagnosed in childhood, which may have slightly affected prevalence estimates for the paediatric population.

In conclusion, this registry-based study demonstrates that chILD imposes a significant clinical burden and exhibits considerable phenotypic and clinical variability. Children with DPLD-A presented with symptoms at an earlier age but were diagnosed later and had greater growth impairment than those with DPLD-B, highlighting a substantial gap in timely recognition. Their higher rates of consanguinity and mechanical ventilation underscore the importance of early clinical recognition, genetic evaluation and appropriate respiratory support in this group. Conversely, the higher frequency of airway-related findings in DPLD-B emphasises the need for careful long-term monitoring. Compared with the first chILD-TR report, the registry has expanded substantially in both patient numbers and diagnostic diversity over time, while corticosteroids have remained the most commonly used treatment and the proportion of patients undergoing lung biopsy has decreased. Our findings underscore the vital role of national registry systems in improving our understanding of chILD, informing clinical management and providing a foundation for future prognostic and interventional research.

## Supplementary material

10.1136/bmjresp-2026-004626 online supplemental file 1

10.1136/bmjresp-2026-004626 online supplemental file 2

10.1136/bmjresp-2026-004626 online supplemental file 3

## Data Availability

Data are available upon reasonable request.
