# Supplementary material for: Current status of childhood interstitial lung disease in Turkiye: 5-year data from ChILD-TR
Source: BMJ Open Respir Res. 2026 Nov 2;13(1):e004626. doi: 10.1136/bmjresp-2026-004626 (PMC13735306; doi:10.1136/bmjresp-2026-004626)
Supplement: Supplementary file 1 [file bmjresp-13-1-s002.pdf]

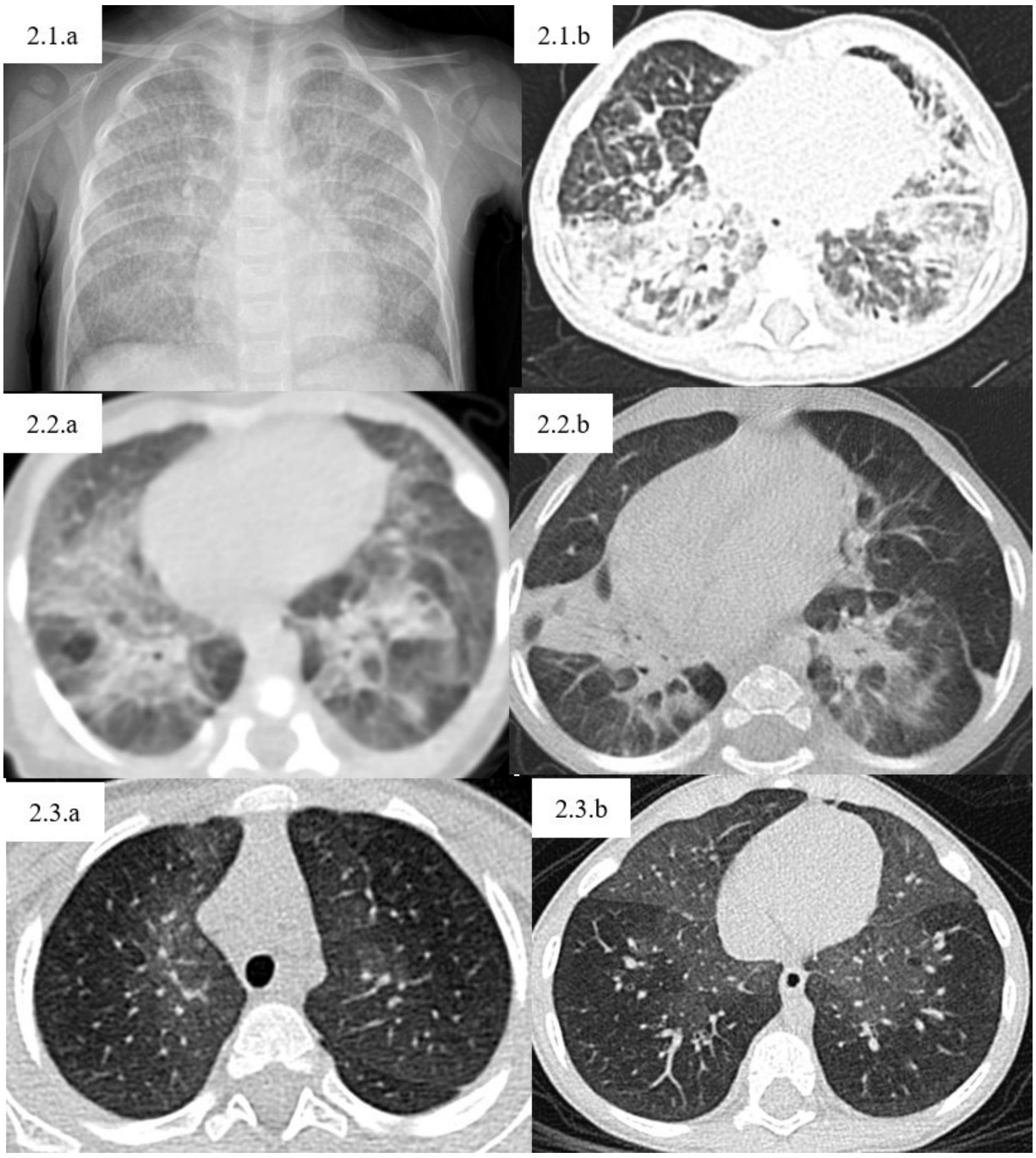

Figure 2. Different radiologic patterns of DPLD-A of chILD

Figure 2.1. A 7-month-old girl with a homozygous MARS1 mutation. **(a)** Chest radiography shows a bilateral diffuse reticulonodular pattern. **(b)** Chest CT reveals diffuse interlobular septal and intralobular interstitial thickening along with ground-glass opacities in both lungs, consistent with pulmonary alveolar proteinosis. Treated with nasal oxygen, a high-calorie, high-protein diet, methionine, and whole-lung lavage. **2.2.** A 45-day-old boy with surfactant protein C deficiency **(a)** Chest CT shows patchy ground glass opacities and increased focal lung aeration between them. Treated with nasal oxygen, steroids, and hydroxychloroquine. **(b)** After 10 months, chest CT reveals regression of ground- glass opacities and atelectatic changes in both lower lobes. **2.3.** An 18-month-old girl with neuroendocrine cell hyperplasia of infancy (NEHI). **(a,b)** Chest CT images show characteristic location of ground-glass opacities for NEHI in **(a)** the medial portion of the upper lobes and **(b)** right middle lobe, lingula, and medial portion of the lower lobes. Treatment was managed with supportive care, including supplemental oxygen, adequate nutrition, and infection control.
DPLD: *diffuse parenchymal lung disease,* ***MARS1:*** *methionyl-tRNA synthetase 1*
