# Supplementary material for: Current status of childhood interstitial lung disease in Turkiye: 5-year data from ChILD-TR
Source: BMJ Open Respir Res. 2026 Nov 2;13(1):e004626. doi: 10.1136/bmjresp-2026-004626 (PMC13735306; doi:10.1136/bmjresp-2026-004626)
Supplement: Supplementary file 2 [file bmjresp-13-1-s003.pdf]

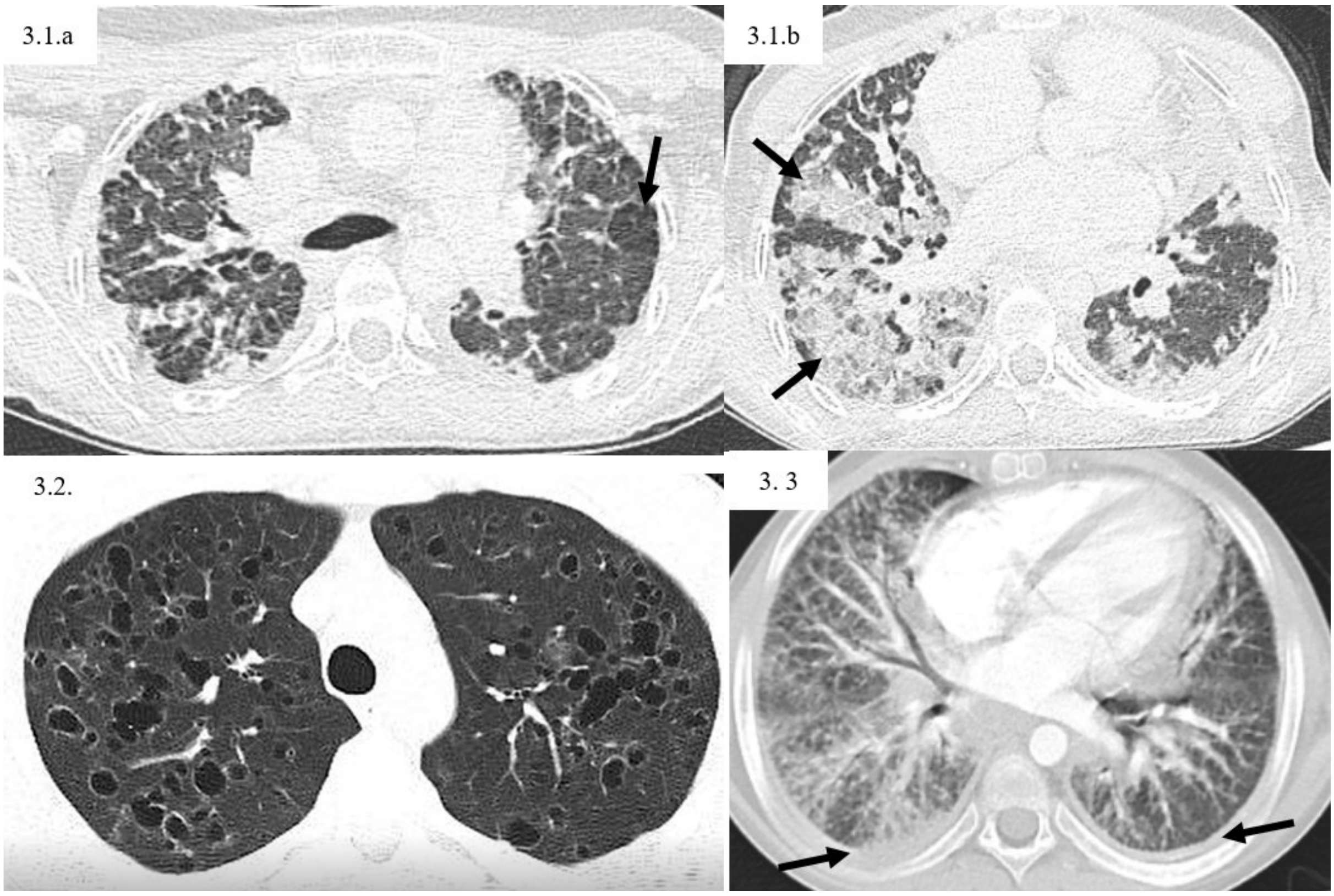


**Figure 3.** Different radiologic patterns of DPLD-B of chILD

**Figure 3.1.** A 20-year-old male with a history of myelodysplastic syndrome and HSCT. Chest CT findings reveal **(a)** bilateral subpleural honeycombing pattern formed by interlobular septal thickening with microcytic changes, a focal area of hyperaeration (arrow) in the upper lobe of the left lung, and **(b)** appearance of crazy-paving pattern **(arrows)** created by diffusely distributed interlobular septal thickening on ground-glass opacities. Findings are consistent with pulmonary fibrosis (PF). He was treated with pirfenidone for PF. **3.2.** A 17-year-old boy with Langerhans cell histiocytosis (LCH). Chest CT demonstrates multiple air-filled cysts of varying sizes in both lungs. The BAL had a 2% CD1a(+) cell, and the cytology of the lung biopsy showed CD1a(+), S100(+), CD68(–), leading to a diagnosis of LCH. He was treated with vinblastine and dexamethasone. **3.3.** A 9-year-old girl was diagnosed with systemic juvenile idiopathic arthritis. Chest CT shows bilateral pleural thickening **(arrows),** ground-glass and reticular opacities in the right lung. No response to routine therapies, and she was treated with a JAK inhibitor, baricitinib.

***DPLD:*** *diffuse parenchymal lung disease,* ***HSCT:*** *hematopoietic stem cell transplantation,* ***JAK****: Janus Kinase*
