# Supplementary material for: Current status of childhood interstitial lung disease in Turkiye: 5-year data from ChILD-TR
Source: BMJ Open Respir Res. 2026 Nov 2;13(1):e004626. doi: 10.1136/bmjresp-2026-004626 (PMC13735306; doi:10.1136/bmjresp-2026-004626)
Supplement: Supplementary file 3 [file bmjresp-13-1-s001.docx]

| **Table 5.** Classification of DPLD Subgroups and Diagnostic Categories in the chILD-TR Registry | | |
| --- | --- | --- |
| **DPLD group** | **Subcategory** | **Diagnostic category** |
| Disorders primarily appearing in infancy (DPLD-A) | DPLD-A1 | Diffuse developmental disorders |
|  | DPLD-A2 | Growth abnormalities / deficient alveolarisation |
|  | DPLD-A3 | Infant conditions of undefined etiology |
|  | DPLD-A4 | Disorders related to the alveolar surfactant region |
|  | DPLD-Ax | Unclear RDS in the mature neonate |
|  | DPLD-Ay | Unclear RDS in the almost mature neonate (30–36 weeks) |
| Disorders that can occur at any age (DPLD-B) | DPLD-B1 | Disorders related to systemic disease processes |
|  | DPLD-B2 | Disorders in the presumed immune-intact host related to exposures (infectious/non-infectious) |
|  | DPLD-B3 | Disorders in the immunocompromised host or transplant recipient |
|  | DPLD-B4 | Disorders related to structural processes of the pulmonary vessels |
|  | DPLD-B5 | Disorders related to reactive lymphoid lesions |
|  | DPLD-Bx | Unclear RDS in the non-neonate |
